# Folic Acid Ionic-Liquids-Based Separation: Extraction and Modelling

**DOI:** 10.3390/molecules28083339

**Published:** 2023-04-10

**Authors:** Alexandra Cristina Blaga, Elena Niculina Dragoi, Alexandra Tucaliuc, Lenuta Kloetzer, Dan Cascaval

**Affiliations:** “Cristofor Simionescu” Faculty of Chemical Engineering and Environmental Protection, “Gheorghe Asachi” Technical University of Iasi, D. Mangeron 73, 700050 Iasi, Romania; elena.dragoi@tuiasi.ro (E.N.D.); alexandra.tucaliuc@academic.tuiasi.ro (A.T.); lenuta.kloetzer@academic.tuiasi.ro (L.K.); dan.cascaval@academic.tuiasi.ro (D.C.)

**Keywords:** CYPHOS IL103, vitamin B9, ionic liquid, extraction, modelling

## Abstract

Folic acid (vitamin B9) is an essential micronutrient for human health. It can be obtained using different biological pathways as a competitive option for chemical synthesis, but the price of its separation is the key obstacle preventing the implementation of biological methods on a broad scale. Published studies have confirmed that ionic liquids can be used to separate organic compounds. In this article, we investigated folic acid separation by analyzing 5 ionic liquids (CYPHOS IL103, CYPHOS IL104, [HMIM][PF_6_], [BMIM][PF_6_], [OMIM][PF_6_]) and 3 organic solvents (heptane, chloroform, and octanol) as the extraction medium. The best obtained results indicated that ionic liquids are potentially valuable for the recovery of vitamin B9 from diluted aqueous solutions as fermentation broths; the efficiency of the process reached 99.56% for 120 g/L CYPHOS IL103 dissolved in heptane and pH 4 of the aqueous folic acid solution. Artificial Neural Networks (ANNs) were combined with Grey Wolf Optimizer (GWO) for modelling the process, considering its characteristics.

## 1. Introduction

**Figure 1:**
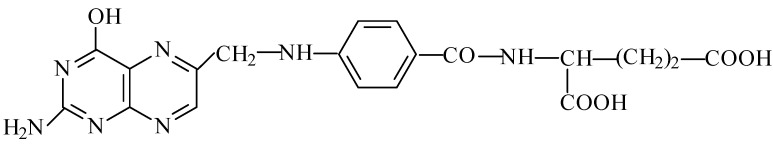
Folic acid (FA) chemical structure.

Vitamins are chemical substances whose derivatives are engaged in the vital metabolic pathways of all living organisms [1]. Since only bacteria, yeast and plants have endogenous routes for vitamin production, humans must obtain most of these crucial nutrients from food [2]. A form of B vitamin called folic acid (folate in its natural form [3])—Figure 1 aids in the maintenance and production of new cells in the body and prevents nucleic acid alterations [2]. Several physiological processes in humans, including the biosynthesis of nucleotides, cell division, and gene expression, as well as the prevention of vascular diseases, megaloblastic anemia, and neural tube defects in developing children, depend on a proper supply of folic acid (FA) or folates.

FA has numerous applications in the pharmaceutical, nutraceutical, food and beverages industries [4], but its stability decreases when exposed to light, moisture, strong acidic or alkaline media, oxygen and high temperatures. It can be found in foods, including oranges, whole-wheat products, dry beans, peas, lentils, oranges, liver, asparagus, beets, broccoli, brussels sprouts and spinach [5].

The estimated value of the global FA market in 2022 was USD 166.8 million. By 2028, it is anticipated to grow to USD 220.9 million, the leading producers being BASF, DSM, Nantong Changhai Food Additive Co., Ltd., Niutang, Zhejiang Shengda, Parchem Fine & Specialty Chemicals, Xinjiang Wujiaqu Xingnong Cycle Chemical Co., Ltd., Xinfa Pharmaceutical Co., Medicamen Biotech Ltd., Jiangxi Tianxin Pharmaceutical Co., Ltd., and Zydus Pharmaceuticals Ltd. [6].

FA is used as a dietary supplement and is currently produced through chemical synthesis, but this process requires the reduction of ecologically harmful effects. Significant research has been employed to develop microbial strains (*Ashbya gossypii* ATCC 10895, *Lactococcus lactis* NZ9000, *Bacillus subtilis* 168) in order to manufacture FA [7,8,9,10]. The highest production value to date has been recorded for *A. gossypii*, which can synthesize 6.59 mg/L of folic acid after metabolic engineering (from its natural ability to produce 0.04 mg/L) [11]. For this process to be successfully employed, efficient separation methods must be developed. More research is required to create a method that is both economical and environmentally benign for producing high-purity FA, because its industrial separation involves many expensive downstream steps and requires mild conditions due to acid’s instability.

Various technologies can be used to separate carboxylic acids (ion exchange, electrodialysis, ultrafiltration, solvent extraction, and membrane processes) [12]. However, reactive extraction using particular extractants has been proven to be an excellent alternative to classical methods, due to its many advantages [13]. Reactive extraction is based on a reaction between an extractant (dissolved in the organic solvent) and the target solute (e.g., carboxylic acids dissolved in the aqueous phase). Several carboxylic acids (gallic acid [14,15], keto-gluconic acid [16], pseudo-monic acid [17], lactic acid [18]), and vitamins (vitamin C [19], vitamin B5 [20]) have been successfully separated through this method at laboratory scale. For sustainability of this process, finding a selective, affordable, and effective extractant and diluent system based on maximal efficiency and minimal toxicity and determining the ideal implementation circumstances are the key challenges in using reactive extraction for the recovery of organic acids. Several characteristics must be considered for the choice of the organic phase, such as selectivity, solubility, cost and operational safety, hydrophobicity, density, polarity, viscosity, recoverability and environmental effects (the use of volatile organic solvents harms the environment). Thus, based on their superior characteristics, ionic liquids (tunable organic salts obtained as a combination of an organic cation and either an organic or a polyatomic inorganic anion in a liquid state below 100 °C) are effective alternatives to classical solvents. The use of most ionic liquids has several advantages over organic solvents, and they play crucial roles in the extraction processes; high thermal stability, negligible vapor pressure, and biocompatibility make them environmentally friendly substances with excellent solvation ability. Based on their properties, most ionic liquids can be employed in green chemistry concepts [21,22]. Lactic acid, citric acid, mevalonic acid [23], and butyric acid [24] have all been successfully extracted using ionic liquids. For the micro-solid phase extraction (for preconcentration and analysis) of pyridoxine and folic acid from biological samples, Zare et al. (2015) investigated a sorbent obtained through the synthesis of gold nanoparticles (Au NPs) and their subsequent transfer to aqueous solution by the application of the ionic liquid: 1-hexyl-3-methylimidazolium bis(trifluoro-methyl-sulfonyl)imide [25].

For the scale-up application of reactive extraction, rigorous modelling and optimization of laboratory-scale studies are essential and different models can be applied [15,16,17]. ANNs are inspired by the biological brain, and GWO is inspired by the grey wolf’s social hierarchy and hunting mechanism [26]. In this work, ANN represents the process model, while GWO is used for model optimization. This combination was considered based on the difficulties in identifying the optimal characteristics of an ANN for a given problem. From the multitude of ANN types, the fully connected feed-forward multilayer perceptron was selected because it is well suited to the complexity and characteristics of the studied process. Moreover, this type of network was successfully applied to solve different problems, including modeling of phytocompounds extraction from dragon fruit peel [27], pectinase extraction from cashew apple juice [28], and separation of pseudo-monic acids [16]. As a bio-inspired metaheuristic, GWO was efficiently applied to optimize the extraction of essential oil from Cleome Coluteiodes Boiss [29], or biodiesel production from waste oils [30,31].

**Figure 2:**
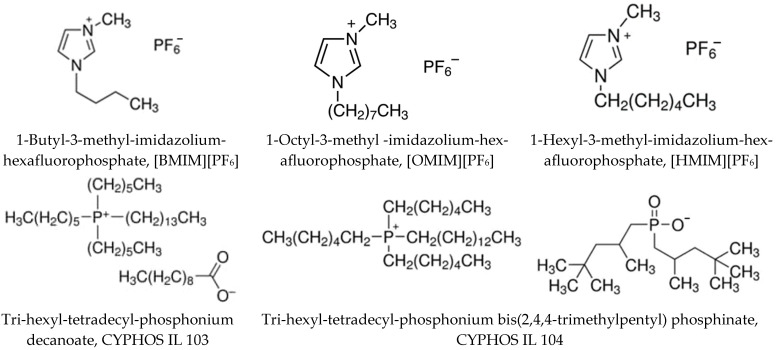
Ionic Liquids (IL) chemical structure.

To the authors’ knowledge, this is the first study regarding folic acid’s separation using ionic liquids (IL) and a liquid–liquid approach. For this research, five ionic liquids (Trihexyl-tetradecyl-phosphonium decanoate, Trihexyl-tetradecyl-phosphonium bis(2,4,4-trimethylpentyl)phosphinate, 1-Butyl-3-methylimidazolium hexafluorophosphate, 1-Octyl-3-methylimidazolium hexafluorophosphate, 1-Hexyl-3-methylimidazolium hexafluorophosphate—Figure 2) were analyzed for FA extraction in order to find an optimal system for the separation process.

Considering the physical characteristics of the ionic liquid (such as high density, viscosity and surface tension) and their high price, three solvents were analyzed as diluents (heptane, chloroform and octanol). The results were discussed from the viewpoint of the extraction mechanism, separation yield and distribution coefficient for different extraction conditions (aqueous phase pH and ionic liquids concentration in the organic phase). Supplementary Artificial Neural Networks (ANNs) were combined with Grey Wolf Optimizer (GWO) to model the considered process.

## 2. Results and Discussions

### 2.1. Extraction Process

Liquid–liquid extraction is a low-energy separation process with simple technical requirements and gentle operating conditions. Its effectiveness is influenced by several variables, including the type of solute and solvent, the utilized diluent and its physicochemical properties, and the solution pH. For FA extraction 5 ionic liquids with different chemical structures (Figure 2) and properties (Table 1) and 3 organic solvents with different dielectric constants (heptane—1.92, chloroform—4.81 and octanol—10.3 [32]) were investigated. The extraction system was chosen based on its low environmental impact. The ionic liquids chosen are highly hydrophobic [33] and were successfully used for the separation of other carboxylic acids (e.g., lactic acid) [34]. Phosphonium ILs offer, in specific cases and applications, several advantages over other types of ILs, including higher thermal stability, lower viscosity and higher stability in strongly basic or strongly reducing conditions [35].

**Figure 3:**
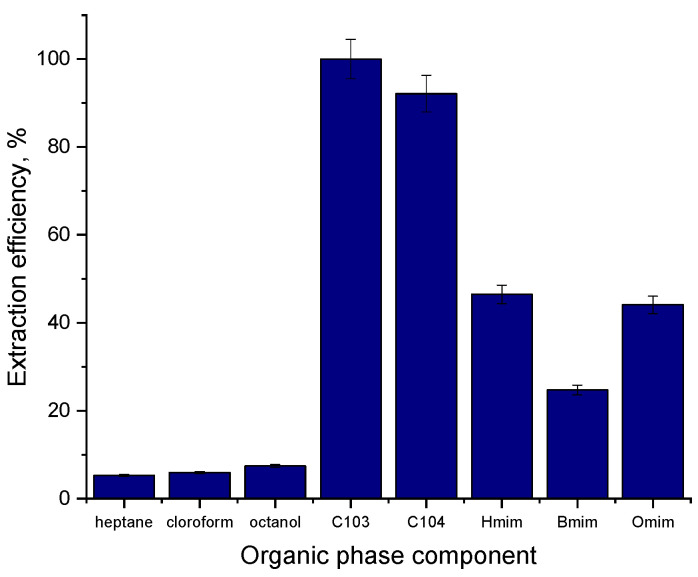
The organic phase composition influence on FA extraction (pH = 4, pure substances).

The results obtained are presented in Figure 3. It can be observed that, without regarding the used organic solvent, the separation yield is very low, proving that physical extraction in classical organic solvents (based only on diffusion and solubilization) is practically impossible for FA. Simultaneously, the ionic liquids can effectively remove FA from the aqueous phase. In general, various variables, including the hydrophobic effect, hydrogen bonding, steric hindrance, and π-π interaction, affect how well ILs can extract carboxylic acids.

The highest efficiency was obtained for quaternary phosphonium salts: CYPHOS IL103 (99.98%) and CYPHOS IL104 (92.85%), the anions decanoate and bis(2,4,4-trimethylpentyl) phosphinate providing significantly superior yields than the hexafluorophosphate $\left[PF_{6}^{-}\right]$ anion, due to stronger hydrogen bonds established between the anion and FA, and to the hydrophobic behavior of the trihexyl(tetradecyl)phosphonium cation (phosphonium IL possesses the highest hydrophobicity among ILs [34]), present in both CYPHOS IL103 and CYPHOS IL104 structure). The superior efficiency that was obtained using CHYPHOS IL103 can be explained by the effect of both the interference of sterically hindrance in CYPHOS IL104 case during the chemical reaction (CYPHOS IL104 has a larger structure compared to CYPHOS IL103 due to the presence of bis(2,4,4-trimethylpentyl)phosphinate ion compared to decanoate anion) and the superior viscosity of CYPHOS IL104 (Table 1)—according to the Wilke-Chang equation, diffusivity varies inversely with viscosity [34]. Similar results (superior values for CYPHOS IL103 compared with other ionic liquids) were obtained by Schlosser et al., 2018, for lactic and butyric acids [38]. These results proved that FA could be successfully separated using ionic liquids; however, their high viscosity and high price are vital points that require more research on this matter.

Due to mass transfer at the liquid–liquid interface, which influences the time required to set the equilibration stage between the aqueous and IL phases, viscosity is a crucial element that affects the kinetics of IL-based extraction systems. Conventional extraction systems using organic solvents may typically reach equilibrium in a short contact time (minutes), whereas IL-based systems require a longer contact time (minutes to hours) due to the high viscosity value, which is in this case (Table 1), between 274 and 805 cP (viscosity of water is 0.89 cP at 25 °C). Because of the influence on the Coulombic interaction between ions, adding an inert solvent could reduce IL viscosity [39]. In this context, the use of heptane, a non-polar solvent, as a diluent to decrease the viscosity and surface tension of the very viscous ionic liquids used, was analyzed as an alternative solution to pure ionic liquids for both CYPHOS IL. For [HMIM][PF_6_], the solvent considered was chloroform, since this ionic liquid and heptane are not miscible. The FA from the aqueous phase can react with the strong hydrophobic ionic liquid dissolved in heptane to generate complexes that are only soluble in the organic phase:

R(COOH)COOH(aq) + [C_14_C_6_C_6_C_6_P][BTMPP]→[C_14_C_6_C_6_C_6_P][R(COOH)COO] + BTMPPH

R(COOH)COOH(aq) + [C_14_C_6_C_6_C_6_P][CH_3_(CH_2_)_8_COO]→[C_14_C_6_C_6_C_6_P][R(COOH)COO] + CH_3_(CH_2_)_8_COOH

The extraction mechanism could be characterized in terms of displacement reaction since ionic liquids are organic salts and contain tri-hexyl(tetradecyl) phosphonium ([C_14_C_6_C_6_C_6_P]) as a cation and bis(2,4,4-trimethylpentyl) phosphinate ([BTMPP]—CYPHOS IL104 and decanoate -CYPHOS IL103) as an anion. The anionic component of the acid (C_13_H_11_N_6_O-CH(CH_2_-CH_2_-COOH)-COO) can displace the anionic species of IL in this reaction (pKa of decanoic acid is 5.7 while folic acid pKa are 4.69; 6.80). The extraction mechanism can also imply hydrogen bonding, the values for hydrogen-bonding interaction energy in the equimolar cation-anion mixture (E_HB_/(kJ/mol) are −38.64 for decanoate [C_9_H_20_CO_2_]^−^ and −38.45 for Bis(2,4,4-trimethylpentyl)phosphinate, [C_16_H_34_O_2_P]^−^ [39].

FA is a weak acid in aqueous solutions, stable between pH 2–10, without heating [40], but its maximum stability is in the pH range of 4–10. The aqueous phase pH has a significant effect on extraction efficiency as it controls acid dissociation:

R(COOH)COOH_(aq)_↔R(COOH)COO^−^_(aq)_ + H^+^, pKa_1_ = 4.69

R(COOH)COO^−^_(aq)_↔R(COO^−^)COO^−^_(aq)_ + H^+^, pKa_2_ = 6.8

**Figure 4:**
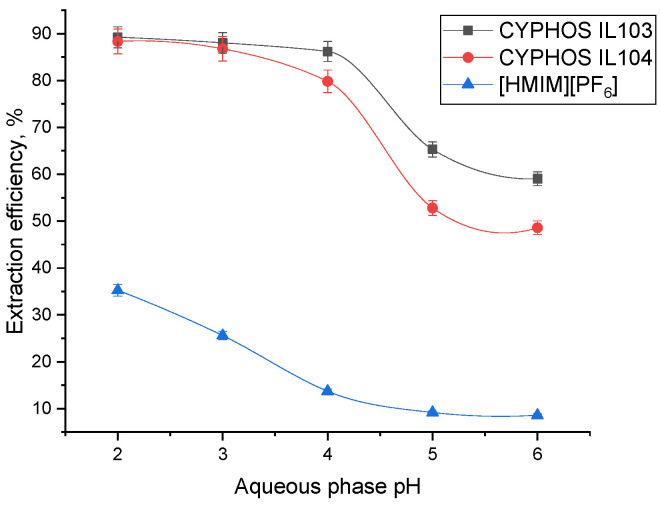
Aqueous phase pH influence on extraction efficiency (ionic liquid concentration 40 g/L).

The extraction efficiency using the purposed extraction system (ionic liquids mixed with an organic diluent) decreases with the increase of aqueous phase pH, as highlighted by the experimental results depicted in Figure 4. Better results were obtained for CYPHOS (dissolved in heptane) compared to [HMIM][PF_6_] (dissolved in chloroform), similar to the results obtained for protocatechuic acid or adipic acid [21]. Due to this fact (extraction efficiency much lower for [HMIM][PF_6_]), the ionic liquid concentration influence was only analyzed for CYPHOS IL103 and CYPHOS IL104.

The experimental results proved that FA could only be extracted by CYPHOS IL103 and CYPHOS IL104 in its undissociated state through H-bond coordination. FA is present in the aqueous solution in an undissociated form at pH lower than 4.5, as determined by the pKa values of 4.69 for the first carboxylic group and 6.80 for the second carboxylic group. This supports the idea that FA will be reactively extracted utilizing a coordination mechanism similar to lactic acid extraction, using CYPHOS IL104 [41]. Furthermore, the results prove that satisfactory separation efficiency can be achieved even at pH equal to 4, at which FA stability is considered maximum.

**Figure 5:**
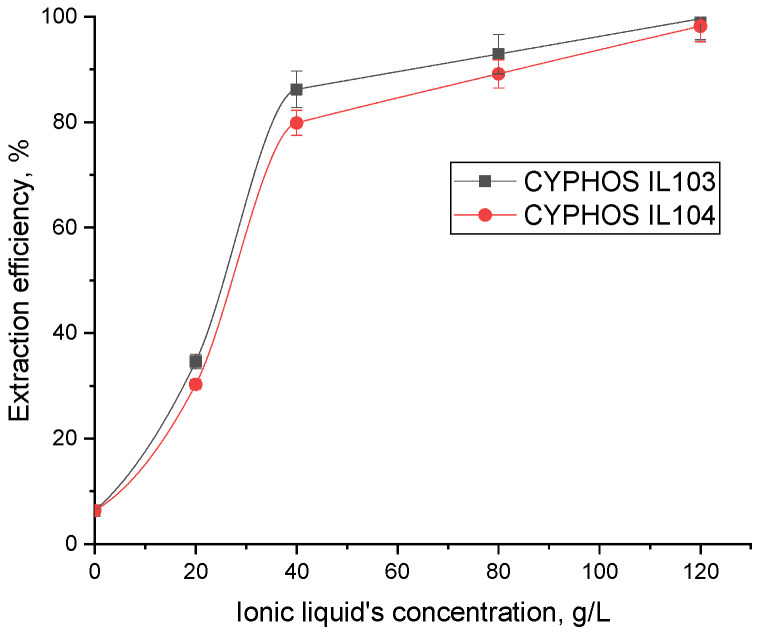
Ionic liquid concentration influence on extraction efficiency (initial phase pH = 4).

The extraction efficiency of FA increases with IL (CYPHOS IL103 and CYPHOS IL104) concentrations in heptane, as seen in Figure 5, proving that this parameter has a critical impact on extraction efficiency. This variation is due to the increase of one reactant concentration at the reaction interface; a higher concentration of IL is more likely to extract a higher concentration of the targeted folic acid from the aqueous phase. No third phase formation was observed in the experiments.

The influence of ionic liquids concentration on extraction efficiency was analyzed at pH 4 to avoid denaturation [40]. In order to establish how many molecules of acid and ionic liquid are involved in the formation of the interfacial complex, the loading factor, Z, defined as the ratio between the concentrations of FA and ionic liquid in the organic phase: [FA]org/[IL]org, was calculated (Table 2).

Two cases can be considered when the variation of the Z parameters is assessed [42]. The first implies that, as the concentration of the ionic liquid increases, so do the loading factor values. This phenomenon, known as overloading (loading larger than unity), shows the formation of complexes with more than one acid per ionic liquid molecule. The maximum value obtained for the loading factor was 0.53 and 0.57 for both ionic liquids, so no overloading was noted in the system. The second case implies that complexes include more than one ionic liquid molecule if the loading factor values decrease as the IL concentration rises. The obtained results, presented in Table 2, showed that for ionic concentrations below the value equal to 40 g/L (0.05 M and 0.06 M for CYPHOS IL104 and CYPHOS IL103, respectively), the loading ratio slowly increases (from 0.43 to 0.53/0.57) between the concentrations 0.03 M and 0.06 M (20–40 g/L), followed by a significant decrease of Z with the increase in ionic liquid concentration. Thus, two types of complexes are formed in direct connection with the ionic liquid concentration:

- For concentrations lower than 40 g/L, echi-molecular complexes (involving only one molecule of both FA and ionic liquid) for both cases: [FA][CYPHOS IL103] and [FA][CYPHOS IL104] are formed, while
- For concentrations higher than 40 g/L, the decrease of Z with increasing ionic liquid concentration in the organic phase indicates the formation of complexes involving two molecules of ionic liquid per folic acid molecule: [FA][CYPHOS IL103]_2_ and [FA][CYPHOS IL104]_2_. This suggests that it may be more cost-effective to enhance the extractant concentration when the process efficiency is below the optimum level than to raise the process efficiency while maintaining the extractant concentration [42].

Schlosser [37] described two methods for water coextraction in the organic phase using Cyphos IL-104 dissolved in dodecane: the production of reverse micelles, and the incorporation of water into hydrated complexes including lactic acid and IL, complexes that include two molecules of water. In this study, no modifications of the two phases volume were recorded after the extraction using heptane as an organic solvent. This could be due to the short extraction time—10 min, or to the large structure of folic acid and its lower concentrations than the ionic liquid.

### 2.2. Modeling

The proposed ANN-GWO approach was applied to model the process. To reach this objective, the extraction yield for FA was determined as a function of the type of solvent used, aqueous phase pH, type of extractant and its concentration. Since the type of solvent and type of extractant are categorical values, they were coded with numerical integer values. This strategy allowed the development of a single model for all possible combinations regarding the considered parameters. This represents one significant advantage over the classical regression methods usually used to determine a process model.

Since the experimental work aimed to perform a reduced number of experiments and since ANNs work better with large datasets, in this work the experimental data were supplemented with a series of additional points by performing an interpolation procedure for simple, individual cases of combinations of parameters. For these cases, based on 2D plot representation, the trendlines that best fit the experimental points were determined using the R^2^ metric, and the identified equation was then used to generate additional data. In this manner, the available dataset was extended from 27 experimental points to 103, thus allowing the ANN model to better capture the dynamic and influence of all process parameters on the extraction yield. Next, following the standard procedures regarding the application of ANNs, the data was normalized and randomly split into two groups (training and testing). The normalization procedure scales all the parameters to the [−1,1] interval and reduces the impact of inputs with higher orders of magnitude. The type of normalization considered in this work is the Min-Max approach [43]. Afterward, the entire available dataset was randomly attributed: 75% to training and 25% to testing. The statistical indicators for these two subsets are presented in Table 3.

In the next step, the limits for the maximum ANN topology and the settings for GWO were set. Regarding topology, to reduce the number of ANN parameters that need to be identified based on the available data, the maximum number of hidden layers was set to 1, with 20 neurons in the hidden layer. This limitation was based on a set of preliminary runs that indicated that, for the current process, an ANN with a single hidden layer could efficiently capture the system’s dynamic. Concerning the GWO parameters, the population size was set to 50 individuals, and the number of runs was set to 500. Next, 50 simulations were performed to determine the best ANN model for the process. The statistics of these runs are presented in Table 4, where Fitness measures model efficiency and is determined based on the Mean Squared Error (MSE) obtained in the training phase. The ANN with the highest fitness, referred to as ANN (4:05:01), indicates the best model for the process. In Table 4, topology is represented using an Input:HiddenLayer:Output notation, where Input represents the number of inputs corresponding to the process parameters (type of solvent used, aqueous phase pH, type of extractant and its concentration), HiddenLayer indicates the number of neurons in the hidden layer and Output indicates the number of process outputs (which for the current problem is FA).

**Figure 6:**
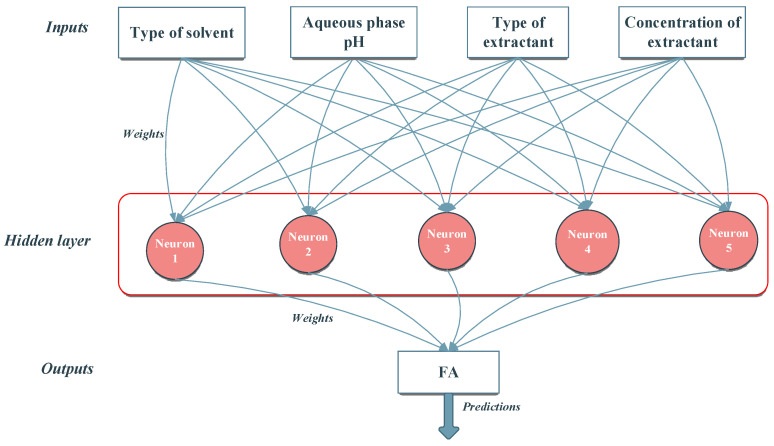
Topology of the best ANN model obtained.

The average absolute error computed in the training phase for ANN (4:05:01) presented in Figure 6 was 6.8%, and in the testing phase was 3.8%. In Figure 6, the hidden layer contains in total 5 neurons, numbered Neuron1 through Neuron5.

These results and the low values for MSE (Table 4) indicate the selected model’s performance. One explanation for the MSE in the training phase being higher than in the testing phase is related to the fact that it contains the only two examples in the entire dataset with a 0 value for the extractant concentration. Only for these two cases is the absolute error high (~50%), with an experimental value of 6.2 and predicted values of ~9.2.

**Figure 7:**
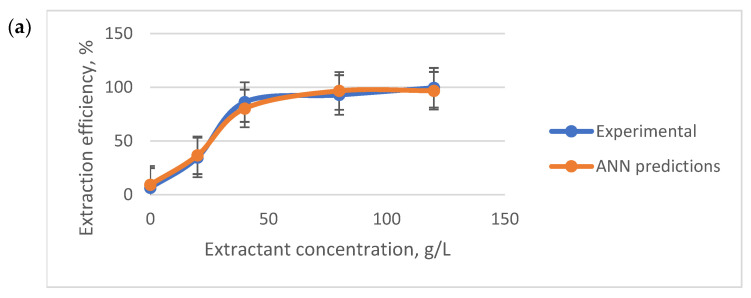

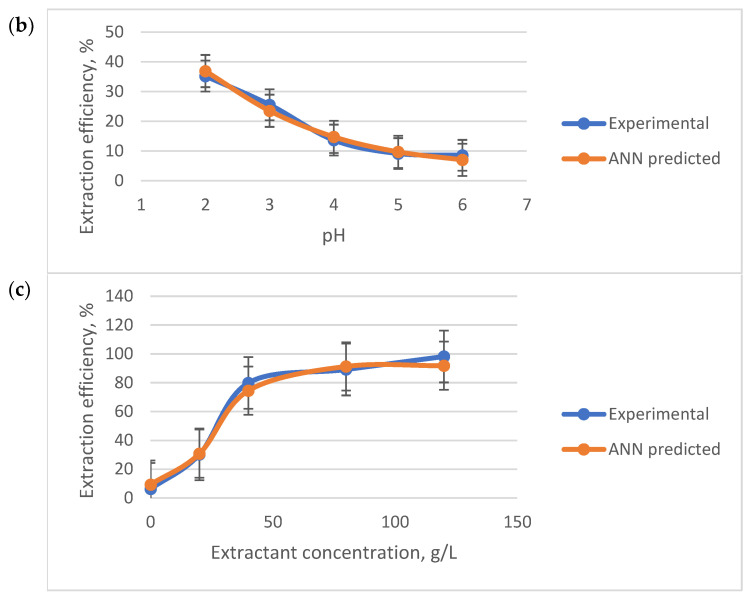
Comparison between the experimental data and the ANN predictions for (**a**) Heptane and CYPHOS IL103 when pH = 4; (**b**) chloroform and [HMIM][PF_6_] when extractant concentration is 40; (**c**) Heptane and CYPHOS IL104 when pH = 4.

An analysis of the data obtained in laboratory settings showed that the identified ANN captures well the system’s behavior in different combinations of solvent-extractant (Figure 7a–c). The relations describing the model are presented in the Supplementary Material.

## 3. Materials and Methods

### 3.1. Extraction Process

The experiments performed for FA extraction were carried out using a vibration shaker (WIZARD IR Infrared Vortex Mixer, VELP Scientifica Srl, Usmate (MB), Italy) that ensured a stirring speed of 1200 rpm (extraction time—10 min and temperature 22 °C), using equal volumes (2 mL) of FA solution, and the organic phase using a glass cell. FA was extracted from aqueous solutions whose initial concentration was 0.04 g/L (due to limited solubility in water). The extraction was carried out either using only pure ionic liquids (preliminary studies): [BMIM][PF_6_]: 1-Butyl-3-methylimidazolium hexafluorophosphate, [HMIM][PF_6_]: 1-Hexyl-3-methylimidazolium hexafluorophosphate, [OMIM][PF_6_]: 1-Octyl-3-methylimidazolium hexafluorophosphate, CYPHOS IL103—Trihexyl-tetra-decyl-phosphonium decanoate, and CYPHOS IL104—Tri-hexyl-tetra-decyl-phosphonium bis(2,4,4-trimethylpentyl)phosphinate, or organic solvents with different dielectric constants (heptane, chloroform and octanol) and a mixture of ionic liquid and organic solvent (CYPHOS IL103 and heptane, CYPHOS IL104 and heptane and [HMIM][PF_6_] and chloroform). All reagents were procured from Sigma-Aldrich [Merck KGaA, Darmstadt, Germany]. The ionic liquid concentration in the organic phase varied between 0 and 120 g/L. The pH of the initial aqueous phase was corrected to the predetermined value, using 4% sulfuric acid and sodium hydroxide solutions, based on the indications of a Hanna Instruments pH 213 digital pH meter (Woonsocket, Rhode Island). After extraction, the samples were separated by centrifugation at 4000 rpm for 5 min, using a DLAB centrifuge (Beijing, China).

The analysis of the acid extraction process was carried out using the separation yield. It was calculated by determining the FA concentration from the initial solution and the raffinate solution, using a Dionex Ultimate HPLC system (Thermo Fisher Scientific Inc., Waltham, MA, USA) equipped with an Acclaim PA2 column, the mobile phase being a mixture of acetonitrile and 30 mM KH_2_PO_4_ solution with a flow rate of 0.5 mL/min, detection at 270 nm. All experiments were performed in triplicate (n = 3, error between 1.5 and 4.5%).

### 3.2. Modeling

Combining mathematical modeling with experimental research can offer a tool for anticipating extraction performance. In order to extend experimental knowledge, the FA separation process was modelled. The methodology applied in this work combined ANNs with the GWO algorithm to model the considered process. The role of GWO is to optimize the ANN characteristics. This combination of bio-inspired metaheuristic-ANN belongs to the neuro-evolutive procedures. Neuro-evolution can be applied at different levels: (a) topology; (b) connection weights; (c) learning rule; (d) node behavior [44]. In this work, GWO was applied simultaneously for topology (the number of hidden layers and neurons in each hidden layer), connections’ weights (parameters usually set in the training procedure with algorithms such as Backpropagation) and node behavior (the selection of transfer function and its associated properties) and, to incorporate all these parameters into the structure of real numbers used by GWO, a direct encoding procedure is used. Once this structure is established, the GWO optimizes a population of randomly generated networks until a stop criterion is reached. The stop function is represented by the number of iterations set at the start of the run.

Distinctively from other bio-inspired metaheuristics such as Genetic Algorithms or Differential Evolution, in GWO the population is divided into four groups that simulate the hierarchical structure of wolfs: alpha (the leaders), beta (that supports the alpha), delta (that executes the commands of alpha and beta), omega (that are managed by the delta and are at the bottom of the hierarchy) [45]. The hunting mechanisms that GWO mathematically simulates include: (a) encircling (where the entire pack works together to chase and direct the prey in a strategy to increase the chance of catching it); (b) hunting (movement of wolfs around the prey guided by the alpha); and (c) attacking (attacking the prey when it stopped moving). These principles were implemented in Visual Studio C#, following the mathematical relations described in the work of Mirjalili et al., 2014 [26].

## 4. Conclusions

This study presents the extraction of FA from aqueous solutions using reactive extraction with hydrophobic IL diluted with heptane (a greener alternative to classical solvents [46]) as an efficient technological alternative for this vitamin’s separation. Almost 100% (99.56%) extraction yield was obtained for 120 g/L CYPHOS IL103 and aqueous phase pH equal to 4, the reactive extraction mechanism being based on hydrogen bonding between FA and IL. The analysis of the loading factor indicated no overloading in the extraction systems in optimum conditions. The process was modelled using a combined ANNs with the GWO algorithm; the data obtained showed good accordance between predicted and experimental results. According to the findings of this study, it is possible to use efficiently phosphonium-based ionic liquids for folic acid extraction or reactive extraction. Moreover, the separation procedures were straightforward and free of volatile organic solvents.

## Figures and Tables

**Table 1:** Main ionic liquids’ physical properties used in biosynthetic compounds extraction [21,36,37].

Ionic Liquid	Molecular Formula	mol. wt., g/mol	Viscosity, cP, 25 °C	log P	Toxicity
[BMIM][PF_6_]	C_8_H_15_F_6_N_2_P	284.18	274	4.49	log EC_50_ * = 3.32 μM
[OMIM][PF_6_]	C_12_H_23_F_6_N_2_P	340.29	682	6.05	log EC_50_ * = 2.24 μM
[HMIM][PF_6_]	C_10_H_19_F_6_N_2_P	312.24	585	5.27	log EC_50_ * = 1.25 μM
CYPHOS IL 103	C_42_H_87_O_2_P	665.11	319	14.32	Inhib. ** = 1.5 cm
CYPHOS IL 104	C_48_H_102_O_2_P_2_	773.27	805.8	18.28	Inhib. ** = 2.6 cm

* Determined against *A. fischeri.* ** Determined against *Shewanella* sp. (inhibition zone, ±0.2).

**Table 2:** Loading factor and distribution coefficient values obtained for C103 and C104 in heptane.

	CYPHOS IL103 conc., M	D	Loading Factor	CYPHOS IL104 conc., M	D	Loading Factor
1	0.03	0.52	0.43	0.02	0.43	0.43
2	0.06	6.24	0.53	0.05	3.95	0.57
3	0.12	12.99	0.28	0.10	8.19	0.32
4	0.18	231.18	0.20	0.15	53.51	0.23

**Table 3:** Statistics descriptors for the subsets used for ANN training and testing.

Subset	Indicator	Type of Solvent	Type of Extractant	Aqueous Phase pH	Extractant Concentration	FA
Training	Mean	1.155844	1.727273	4.084416	48.7013	63.0724
	Median	1	2	4	40	79.8336
	Standard Deviation	0.365086	0.718851	0.874164	28.2191	32.09486
	Sample Variance	0.133288	0.516746	0.764162	796.3175	1030.08
	Kurtosis	1.792505	−0.94144	0.632868	0.556316	−1.11127
	Skewness	1.935617	0.461642	−0.01714	0.909524	−0.65705
	Minimum	1	1	2	0	6.229
	Maximum	2	3	6	120	98.33493
	Count	77	77	77	77	77
Testing	Mean	1.269231	1.923077	3.75	51.15385	62.5145
	Median	1	2	4	40	81.54103
	Standard Deviation	0.452344	0.796145	0.806226	26.08861	32.47939
	Sample Variance	0.204615	0.633846	0.65	680.6154	1054.911
	Kurtosis	−0.84995	−1.37721	1.646948	0.895838	−1.59354
	Skewness	1.105353	0.143288	−0.10853	1.188646	−0.49504
	Minimum	1	1	2	15	8.340475
	Maximum	2	3	5.75	120	99.5693
	Count	26	26	26	26	26

**Table 4:** Statistic indicators for 50 runs for the ANN-GWO approach.

	Fitness	MSE Training	MSE Testing	Topology
Best	647.7774	0.001544	0.000624	4:05:01
Worst	137.7863	0.007258	0.006111	4:08:01
Confidence interval	318.34 ± 41.06	0.004 ± 0.0005	0.003 ± 0.0004	0.985 ± 0.0018

## Data Availability

Data is contained in the article and Supplementary Material.

## Supplementary Materials

The following supporting information can be downloaded at: https://www.mdpi.com/article/10.3390/molecules28083339/s1, Figure S1: FTIR spectra for folic acid, Cyphos IL103 ionic liquid in heptane and extract; Figure S2: UV-VIS spectra for Cyphos IL103 ionic liquid in heptane and Chyphos Il103 loaded with FA.
