# Supplementary material for: Folic Acid Ionic-Liquids-Based Separation: Extraction and Modelling
Source: Molecules. 2023 Apr 10;28(8):3339. doi: 10.3390/molecules28083339 (PMC10144844; doi:10.3390/molecules28083339)
Supplement: Supplementary file 1 [file molecules-28-03339-s001.zip › molecules-2288050-supplementary.pdf]

This best model obtained for the current process is described by Equations (1)–(11). The relations are written for Python, and can be used to generate predictions for any desired combination of parameters.

$$INP\_0 = -1E - 20 + 0.001 + (SolventType - 1) * (0.999 - 0.001)$$
$$INP\_1 = -1E - 20 + 0.001 + (ExtractantType - 1) * (0.999 - 0.001) /2$$
$$INP\_2 = -1E - 20 + 0.001 + (pH - 2) * (0.999 - 0.001) /4$$
$$INP\_3 = -1E - 20 + 0.001 + (ExtractantConcentration - 0) * (0.999 - 0.001) /120$$
$$H1\_0 = (2.0 / (1.0 + math.exp(-2 * (INP\_0 * 1.81545580728531 + INP\_1 * -0.00314151992726174 + INP\_2 * 1.45781333314899 + INP\_3 * -6.34231656543493 + 0.62639509903911))) - 1.0)$$
$$H1\_1 = math.abs(INP\_0 * -2.8350396545486 + INP\_1 * -0.0200583470773323 + INP\_2 * 0.00112420585586263 + INP\_3 * -0.291646151421369 + -0.0129992139798041)$$
$$H1\_2 = math.abs(INP\_0 * -0.0222387311546859 + INP\_1 * 0.39043851573044 + INP\_2 * -0.237682580329203 + INP\_3 * -0.0849394843588948 + 0.0200096222951101)$$
$$H1\_3 = math.abs(INP\_0 * 0.0258761237759181 + INP\_1 * -1.35934119425144 + INP\_2 * -0.194074280903626 + INP\_3 * -0.0931867089266974 - 0.657576582992829)$$
$$H1\_4 = math.abs(INP\_0 * 9.77206314705546 + INP\_1 * -0.0832814869532355 + INP\_2 * 0.00301007397580383 + INP\_3 * -0.289676388587111 + 0.0184595562102561)$$
$$OUTPUT\_1 = math.abs(H1\_0 * -0.502625950331134 + H1\_1 * 0.0267465377920682 + H1\_2 * -0.0756323296383862 + H1\_3 * -0.0987757102888255 + H1\_4 * 0.00826476558196148 + 0.553834190456632) \quad (10)$$
$$Efficiency = (OUTPUT\_1 + 1E - 20 - 0.001) * (99.5693 - 6.229)/ (0.999 - 0.001) + 6.229 \quad (11)$$

To support the extraction equilibrium, UV-VIS spectra of Cyphos IL103 and Cyphos IL103 loaded with FA and FT-IR spectra of folic acid (FA), Cyphos IL103 and Cyphos IL103 loaded with FA are presented in Fig. S1 and S2. Regarding the FTIR analysis, the extract (Cyphos IL103 loaded with FA) spectra shows peaks modifications: at 1608.3 cm^{-1} (shifted from 1606.48 cm^{-1} in FA spectra), at 1405 cm^{-1} (shifted from 1407 cm^{-1} in Cyphos IL spectra), at 1379 cm^{-1} (shifted from 1386 cm^{-1} in Cyphos IL spectra) and at 1638.17 cm^{-1} (present in FA spectra) proved FA presence in the extracted complex (Cyphos IL 103 loaded with FA).

The analysis of the UV-VIS spectra presented in figure S2, shows, for free Cyphos IL103 (red solid line) its maximum UV absorption peaks at 218 nm. For Cyphos loaded with FA (green solid line) the suplementary characteristic UV absorption peak at 280 is visible proving folic acid's presence.

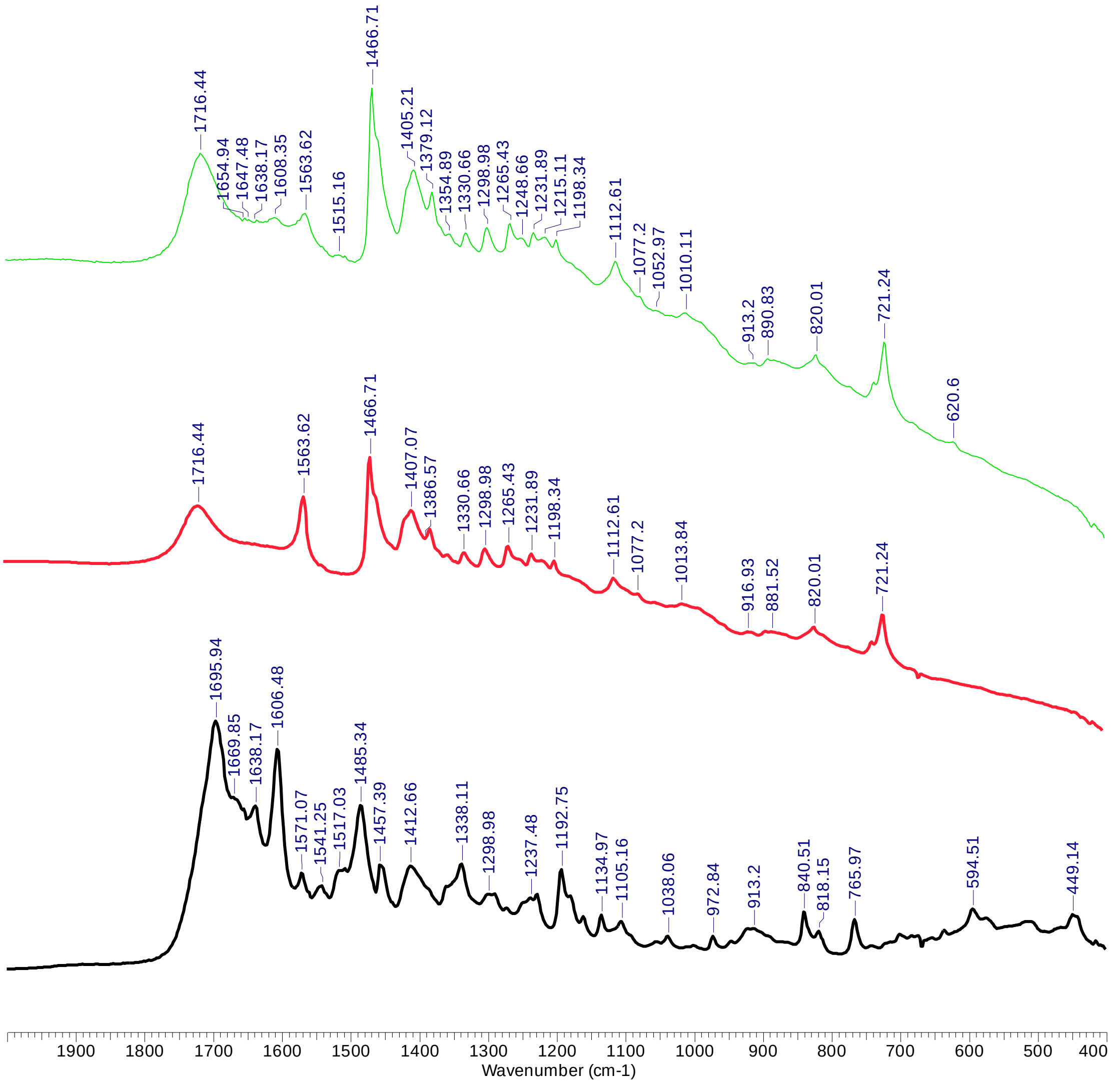

Figure S1. FTIR spectra for folic acid ( ), Cyphos IL103 ionic liquid in heptane ( ) and extract ( ). Potassium bromide (KBr) was used to collect the background, at a weight ratio of 1:100 before the spectrum collection. The FTIR spectra of all samples were determined to be in the range of 500-2000 cm^{-1}, samples were examined at a spectral resolution of 4 cm^{-1} with 32 scans per sample.

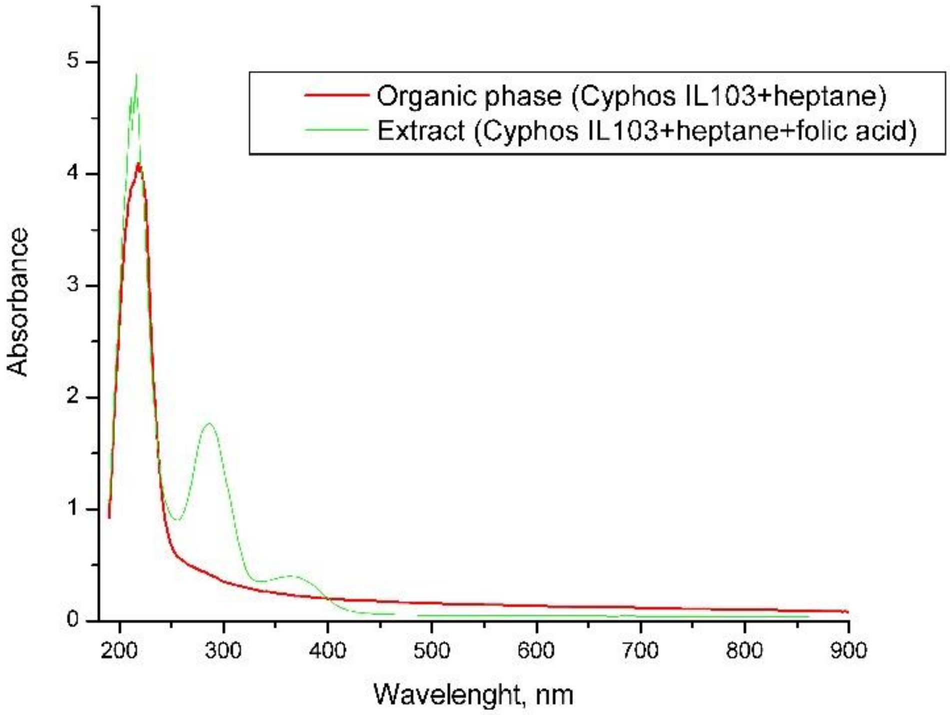

Figure S2. UV-VIS spectra for Cyphos IL103 ionic liquid in heptane ( ) and Chyphos Il103 loaded with FA ( ).
